# Molecular analyses of carangid fish diets reveal inter‐predation, dietary overlap, and the importance of early life stages in trophic ecology

**DOI:** 10.1002/ece3.10817

**Published:** 2024-01-04

**Authors:** Fabricio dos Anjos Santa Rosa, Maria A. Gasalla, Anna Karolina Oliveira de Queiroz, Talita Fernanda Augusto Ribas, Quentin Mauvisseau, Hugo J. de Boer, Birgitte Lisbeth Graae Thorbek, Renato Renison Moreira Oliveira, Marcele Laux, Felippe A. Postuma, Jonathan Stuart Ready

**Affiliations:** ^1^ Group for Integrated Biological Investigation, Center for Advanced Studies of Biodiversity Federal University of Pará Belém Brazil; ^2^ Fisheries Ecosystems Laboratory, Oceanographic Institute University of São Paulo São Paulo Brazil; ^3^ Natural History Museum University of Oslo Oslo Norway; ^4^ Instituto Tecnológico Vale Belém Brazil; ^5^ Postgraduate Program in Bioinformatics Federal University of Minas Gerais Belo Horizonte Brazil; ^6^ Ecology Department Federal University of Minas Gerais Belo Horizonte Brazil

**Keywords:** Brazil, Carangidae, diet analysis, fisheries, metabarcoding

## Abstract

Carangid fishes are commercially important in fisheries and aquaculture. They are distributed worldwide in both tropical and subtropical marine ecosystems. Their role in food webs is often unclear since their diet cannot be easily identified by traditional gut content analysis. They are suspected to prey on pelagic and benthic species, with clupeiform fishes being important dietary items for some species, though it is unknown whether carangids share food resources or show trophic segregation. Here, we used metabarcoding to overcome traditional challenges of taxonomic approaches to analyze the diet of seven carangid species caught as bycatch in the Brazilian southwest Atlantic sardine fishery. Stomach contents were processed from the following species: *Caranx crysos*, *Caranx latus*, *Chloroscombrus chrysurus*, *Hemicaranx amblyrhynchus*, *Oligoplites saliens*, *Selene setapinnis*, and *Trachinotus carolinus*. Identified diets were dominated by teleost fishes. The *C. latus* diet was the most distinct among the seven species, preferentially consuming *Engraulis anchoita*, but *H. amblyrhynchus*, *O. saliens*, and *S. setapinnis* also showed a trend of predominantly consuming small pelagic fishes. Finally, we found evidence of inter‐predation in carangids, especially strong between *S. setapinnis* and *C. crysos*, suggesting that consumption of early life stages may result in indirect competition through reduced recruitment in these fishes. These findings provide unprecedented insights into the biodiversity in marine ecosystems, especially the poorly known diet of carangid fishes.

## Graphical abstract

Metabarcoding of fish stomach contents is a modern approach that can help resolve marine food webs and assist in management of fisheries. Analysis of the diet of seven carangid fish species in the southwest Atlantic using this technique identified principal links that can influence the pelagic sardine fisheries. The contribution of early life stages of other fishes and niche segregation through differential use of the water column are important findings that should help integrate the previously poorly known carangid diets into ecosystem‐based models.

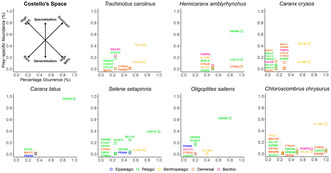

## 1 INTRODUCTION

Optimal foraging theory predicts that behavior patterns guide organisms to ingest more energetic food items (Rogers & Weisler, 2021), prioritizing a small number of preferred prey when resources are abundant (Ratcliffe et al., 2018). In marine fishes, there is evidence that the consumption of diverse items can produce equivalent energy density levels. This maintains stable populations where the quantity of ingestion is more relevant than the quality/type of item (Weitkamp & Sturdevant, 2008). In contrast, the presence of competitors can reduce the number of species and individual niches (Neves et al., 2021; Van Valen, 1965), generating differences in the use of resources (Kinzer & Schulz, 1988; Stasko et al., 2018). This is especially relevant in marine systems where the vertical stratification of the water column generates different levels of resource availability and produces a context in which the type of item can also impact the trophic ecology of these organisms (Neves et al., 2021). The diet of many marine species additionally exhibits a high diversity of small organisms, leading to reduced refinement and resolution in the identification of these food items using traditional taxonomic methods (Dias et al., 2017; Pease et al., 2018). The identification of soft‐bodied organisms, which are often critical components of marine species diets, is especially challenging due to the rapid rate of digestion and fragmentation of such species (Ribas et al., 2021).

Traditional studies in trophic ecology are based on visual identification of food items (Gasalla & Soares, 2001; Medeiros et al., 2017; Soares et al., 1993). However, this method is dependent on the level of digestion of prey and requires a high level of taxonomic knowledge for reliable characterization (Sheppard & Harwood, 2005). In contrast, analyses of taxon diversity in different types of samples can also be made by metabarcoding (Buchner et al., 2021; Hestetun et al., 2021; Leese et al., 2021). This is done by allocating identities through sequence comparison of specific markers to sequences existing in reference databases (e.g., BOLD, GenBank) (Leray et al., 2013). This is a powerful approach for characterizing the diets for diverse groups of organisms using both feces (Chua et al., 2021; Mitchell et al., 2022) and stomach contents (Ando et al., 2018; Kowalczyk et al., 2019; Leray et al., 2013; Shraim et al., 2017; Waraniak et al., 2019). Metabarcoding allows high‐resolution assessments on the diversity and abundance of dietary food items (Nielsen et al., 2017) and wide‐ranging access to the diversity of food items (Bessey et al., 2019; Casey et al., 2019; Cowart et al., 2015) through relative comparisons of abundance (Ando et al., 2020; Deagle et al., 2019; Kartzinel et al., 2015). This provides data that are relevant for both biodiversity studies and management policies since they supply information on necessary habitat components for species conservation (Ficetola et al., 2018). Under optimal conditions, relative read abundance can also provide information on feeding preferences and dominant components of diet (Berry et al., 2015; Deagle et al., 2019).

The role of carangid fishes in food webs is unclear as their diet cannot be easily identified in traditional gut content analysis. It is important to correctly identify trophic relationships in food webs to inform ecosystem‐based management of fisheries (Gaichas et al., 2010), but few studies have evaluated the diet of more than one or two species at a time (Barreiros et al., 2003; Carvalho & Soares, 2006; Jardas et al., 2004; Velasco‐Reyes et al., 2022; Yankova et al., 2008; Young & Davis, 1992), and the influence of inter‐species competition on carangid diets is currently unknown. Clupeiform fishes are known to be important dietary items for some carangid species, but they also prey on a range of pelagic (Deudero, 2001) and benthic species (Blaber & Cyrus, 1983; Palmeira & Monteiro‐Neto, 2010). There are very few studies on the diets of Carangidae caught in the same location (Blaber & Cyrus, 1983; Deudero, 2001; Palmeira & Monteiro‐Neto, 2010; Silvano, 2001; Velasco‐Reyes et al., 2022), and we do not currently understand whether species in this family share food resources or show niche segregation. Mouth position in fishes is correlated with predatory mode, with some species superior (or supra‐terminally) opening mouths indicating consumption of prey from above them, while others have terminal opening mouths to feed on prey in the same stratum of the water column and others have inferior (or sub‐terminally) opening mouths that are associated with predating items from below them in the water column (Facey et al., 2022). Carangids vary in their use of strata in the water column and in terms of mouth morphology, with superior opening mouths in some genera (e.g., *Chloroscombrus*, *Oligoplites*, and *Selene*) and others with terminal (e.g., *Caranx* and *Hemicaranx*) or inferior (e.g., *Trachinotus*) opening mouths (Palmeira & Monteiro‐Neto, 2010; Velasco‐Reyes et al., 2022).

Here, we used metabarcoding to understand the trophic diversity of seven species of carangids caught as bycatch in the Brazilian southwest Atlantic sardine fishery. We aim to identify possible patterns of exploration of the water column based on the stratum in which the food items are usually found, and to understand how the type of foraging behavior may affect the diet of these species.

## 2 MATERIALS AND METHODS

### 2.1 Sampling

Samples were collected between March and September 2016 during on‐board fishery monitoring of the pelagic sardine fishery in south‐eastern Brazil (between Barra Velha, Santa Catarina state and Rio de Janeiro, Rio de Janeiro state) as part of a regional ecosystem‐based fisheries management project based at the University of São Paulo Oceanographic Institute. Samples came from five capture events from three fishing embarkations of purse seine fishing vessels (two capture events from one embarkation just west of Ilha Grande, Rio de Janeiro, two capture events from one embarkation just east of Ilha Grande, Rio de Janeiro, and one capture event from an embarkation at Barra Velha, Santa Catarina—metadata in Ready et al., 2023). These purse‐seine caught samples are ideal for metabarcoding analyses as they have not consumed bait and have little time to eat co‐collected taxa during their capture (Ribas et al., 2021). Nonetheless, records were also made of co‐collected taxa to control for their possible influence on dietary findings. We collected as many individuals as possible from each species identified in the bycatch, including the carangid fishes: *Caranx crysos* (Mitchill, 1815) (*N* = 7); *Oligoplites saliens* (Bloch, 1793) (*N* = 5); *Chloroscombrus chrysurus* (Linnaeus, 1766) (*N* = 4); *Selene setapinnis* (Mitchill, 1815) (*N* = 4); *Trachinotus carolinus* (Linnaeus, 1766) (*N* = 4); *Caranx latus* Agassiz, 1831 (*N* = 3); and *Hemicaranx amblyrhynchus* (Cuvier, 1833) (*N* = 2) (Table 1). Whole fishes were frozen until treatment in the laboratory at the University of São Paulo—USP. Samples and equipment were decontaminated prior to opening the fishes and removing their stomach contents. The contents were preserved in absolute ethanol in decontaminated pots with external labelling and transferred to the Federal University of Pará—UFPA for further processing. Samples were collected under SISBIO license number 53022‐2 from the “Instituto Chico Mendes de Conservação da Biodiversidade.” Work was performed under approval of the UFPA Ethics Committee (CEUA‐UFPA—Permit 68/2015).

**TABLE 1:** Sample data for stomach contents of seven Carangid fish species collected as part of bycatch of the southern Brazilian sardine fishery.

Species	Sample code (# subsamples)	Length (mm)
*Caranx crysos*	CCY86 (4 – a, b, c, d)	159
*Caranx crysos*	CCY88 (4 – a, b, c, d)	165
*Caranx crysos*	CCY180 (4 – a, b, c, d)	173
*Caranx crysos*	CCY181 (4 – a, b, c, d)	169
*Caranx crysos*	CCY182 (4 – a, b, c, d)	171
*Caranx crysos*	CCY217 (2 – c, d)	150
*Caranx crysos*	CCY220 (2 – a, d)	165
*Caranx latus*	CLA1 (4 – a, b, c, d)	145
*Caranx latus*	CLA5 (3 – a, b, d)	153
*Caranx latus*	CLA10 (4 – a, b, c, d)	142
*Chloroscombrus chrysurus*	CCH333 (3 – a, c, d)	238
*Chloroscombrus chrysurus*	CCH334 (3 – a, c, d)	245
*Chloroscombrus chrysurus*	CCH335 (4 – a, b, c, d)	251
*Chloroscombrus chrysurus*	CCH336 (4 – a, b, c, d)	235
*Hemicaranx amblyrhynchus*	CCY337 (2 – c, d)	333
*Hemicaranx amblyrhynchus*	CCY338 (4 – a, b, c, d)	285
*Oligoplites saliens*	OSA282 (3 – a, b, c)	225
*Oligoplites saliens*	OSA283 (4 – a, b, c, d)	281
*Oligoplites saliens*	OSA284 (4 – a, b, c, d)	245
*Oligoplites saliens*	OSA285 (4 – a, b, c, d)	249
*Oligoplites saliens*	OSA332 (4 – a, b, c, d)	332
*Selene setapinnis*	SSE236 (2 – c, d)	151
*Selene setapinnis*	SSE237 (2 – c, d)	151
*Selene setapinnis*	SSE238 (2 – c, d)	151
*Selene setapinnis*	SSE239 (2 – c, d)	150
*Trachinotus carolinus*	TCA240 (2 – c, d)	151
*Trachinotus carolinus*	TCA241 (2 – c, d)	152
*Trachinotus carolinus*	TCA253 (2 – b, d)	199
*Trachinotus carolinus*	TCA254 (3 – b, c, d)	174

*Note*: Sample codes are the same as indicated in the GenBank deposited data (Study accession ID SRP269542).

### 2.2 DNA extraction, PCR, and sequencing

All procedures were performed after decontamination of all materials and surfaces using bleach and/or UV light exposure. Individual stomach contents were separated from preservative by three cycles of centrifugation, washed with ultrapure and UV sterilized water and homogenized. Four 650 μL subsample replicates (a–d) were separated and stored in separate microcentrifuge tubes. DNA from all replicates was extracted using the CTAB/phenol/chloroform protocol (Doyle & Doyle, 1987) in a decontaminated fume hood. Sixty subsamples representing the 21 samples of carangid fishes were successfully extracted and sequenced (successful amplification of replicates a–d is indicated in Table 1 as some replicates did not amplify, apparently because of PCR inhibition), as well as multiple negative controls. These controls were generated at various steps, including sample processing (tubes opened and filled only with ethanol or water during sample preparation—one control between each homogenized sample), DNA extraction (one negative control in each row of 12 tubes) and amplicon production (a minimum of two spatially separated negative controls on each PCR plate).

PCR amplifications of all subsamples and negative controls targeting the Cytochrome C oxidase subunit I marker (130 bp) were performed using the primers Minibar‐Mod‐F and Minibar‐Mod‐R (Berry et al., 2015), following index combinations as described in Fadrosh et al. (2014). PCRs were carried out in 25 μL volumes with 1× Q5 High‐Fidelity master mix (New England Biolabs), 1× Q5 enhancer (New England Biolabs), 0.5 μM of each primer, and 2–3 ng of template DNA (including unique combinations of dual‐indexing barcodes). The annealing temperature was 55°C and the thermal profile was as recommended by the supplier.

Amplicons were visualized on agarose gels and quantified using ImageLab Software v6.0. (Bio‐Rad Laboratory). Based on this quantification, amplicons were normalized using a Biomek 4000 liquid handling robot (Beckman Coulter). The DNA library was cleaned using 1.0× AMpure beads (Beckman Coulter), and sequence adapters ligated to the dual‐indexed amplicons using the NEBNext Fast DNA Library Prep Set for Ion Torrent (New England Biolabs). The amplified libraries were size selected using BluePippin (Sage Science). The final libraries were quantified on a Fragment Analyzer (Agilent) instrument using the High Sensitivity Genomic DNA Kit (Agilent) and sequenced on two 530 chips on an Ion GeneStudio S5 system (Thermo Fisher).

### 2.3 Bioinformatics

We used the pipeline PIMBA (Oliveira et al., 2021) to process the sequencing data. The raw sequencing data were first demultiplexed using the dual‐index barcodes. The demultiplexed FASTQ files were then cleaned to remove low‐quality bases (PHRED < 20) using PRINSEQ (Schmieder & Edwards, 2011), with the *pimba_prepare* module. Then, we used QIIME (Caporaso et al., 2010) and VSEARCH (Rognes et al., 2016) pipelines in the *pimba_run* module to perform dereplication, discard singletons, trim sequences to 130 bases, remove chimeric sequences, and cluster molecular operational taxonomic units (MOTUs) using similarity thresholds of 97% and 99%. Also, with *pimba_run*, MOTUs were aligned with the *nt* reference database from NCBI using BLAST to perform taxonomic assignment, including the use of LULU (Frøslev et al., 2017) to remove erroneous MOTUs and PIMBA script to check for and exclude MOTUs representing nuclear mitochondrial pseudogenes (NUMTs) based on descriptors considering the 10 most similar sequences identified by BLAST. Assignment was performed using minimum thresholds of 90% similarity, and similarity to the most similar taxon was recorded. This step was included since extensive reference databases for much of the fauna in the sampled geographic region are not available and we aimed at identifying as many taxa as possible in the diet during post‐processing (see below). The outputs from the different clustering levels were evaluated to determine their effectiveness at recovering taxa without producing multiple OTUs with the same taxonomic assignment.

Rarefaction curves for each replicate were performed to assess whether adequate sequencing depth had been achieved, using random sampling of 999 sequences without replacement in the “rarecurve” function from *vegan* (Oksanen et al., 2020) in R 4.1.0 (R Core Team, 2021).

### 2.4 Post‐processing the data

To remove false positives and possible contaminants or sequencing errors, we applied the following rules: (i) the maximum number of reads detected in the controls was removed for each MOTU from all samples; (ii) MOTUs containing less than 10 reads were discarded; (iii) obvious non‐target species or MOTUs likely originating from carry‐over contaminations were removed from the dataset (Ushio et al., 2018). The final assignments of dietary items were inferred based on similarity values and knowledge on geographic distributions in the literature accessed through FishBase (Froese & Pauly, 2023) and SeaLifeBase (Palomares & Pauly, 2022) to confirm the taxonomic identification. Species identity was considered confirmed for similarities above 97%, and the most probable geographically local representatives of the same genus or family identified when similarities were between 90% and 97%. For example, a sample assigned with a similarity of 90%–97% could be subsequently assigned at family level, genus level, or even a specific species in the genus known from the region if only one species is known for the region. A minimum of 90% similarity was used, as reassignment based on lower similarities than this becomes unlikely. As no blocking primers were used in this study, the predator's DNA was co‐amplified alongside dietary items. These data were used to confirm predator identification but removed for subsequent dietary analyses.

Detection of prey taxa was normalized, and secondary consumption of items evaluated by using thresholds of relative read abundance (RRA) within samples (<1% for exclusion and 1%–5% as a flag to assess secondary consumption). Flagged items were then evaluated using comparative data from the literature (Blaber & Cyrus, 1983; Carvalho & Soares, 1997, 2006; Cunha et al., 2000; Jardas et al., 2004; Medeiros et al., 2017; Palmeira & Monteiro‐Neto, 2010; Sazima, 1998; Silvano, 2001; Sley et al., 2009; Smith‐Vaniz, 1986; Velasco‐Reyes et al., 2022; Yankova et al., 2008; Young & Davis, 1992) and ongoing metabarcoding analysis of dietary items of fish (the same species identified as principal carangid prey items in this study, i.e., those with RRA > 5%) from the same sampling region (Ready, J.S., Rosa, F.A.S., Queiroz, A.K.O., Gasalla, M.A., Ribas, T.F.A., Mauvisseau, Q., de Boer, H.J., Thorbek, B.L.G., Anmarkrud, J.A., Schrøder, A., Oliveira, R.R.M., Laux, M., Postuma, F.A.). Reads were then averaged across subsamples (to avoid inflation of read abundance in samples with more subsamples e.g., 4 vs. 3 vs. 2 subsamples) and identified items then visualized across the samples using a heatmap representing RRA of prey items for MOTUs in each sample (Deagle et al., 2019). Ecological analysis of diet components was then performed using non‐metric multidimensional scaling (nMDS) using both relative abundance (Bray–Curtis) and presence/absence (Jaccard) to assess and confirm the integrity of trends in trophic segregation considering the limitations of this dataset and possible biases in relative abundance analyses in metabarcoding analyses (Lamb et al., 2019). We subsequently applied the *envfit* function to calculate factor averages of prey items (using a Euclidean distance matrix and 999 permutations) in order to define which prey items contributed most to the different groups identified in the nMDS analysis (Borcard et al., 2011). Stress values in nMDS analyses >0.1 indicate some distortion of the data representation (Clarke et al., 2014). The feeding strategy of each species was visualized using Costello graphs (Costello, 1990) as modified by Amundsen et al. (1996). A population in which different individuals specialize on different resource types exhibit a high “between‐phenotype component” (BPC—upper left placement in Costello graphs) as prey items in their diets have high specific abundance but a low percentage of occurrence, but a population in which individuals utilize many resources simultaneously exhibit a high “within‐phenotype component” (WPC—lower right placement in Costello graphs) as many prey items are shared (resulting in a high percentage of occurrence across individuals) but are all consumed at low abundances. All analyses and figures were produced using *ggplot2* (Wickham, 2016) or *vegan* (Oksanen et al., 2020) in R 4.1.0 (R Core Team, 2021). R scripts are available as a file in Ready et al. (2023).

## 3 RESULTS AND DISCUSSION

**FIGURE 1:**
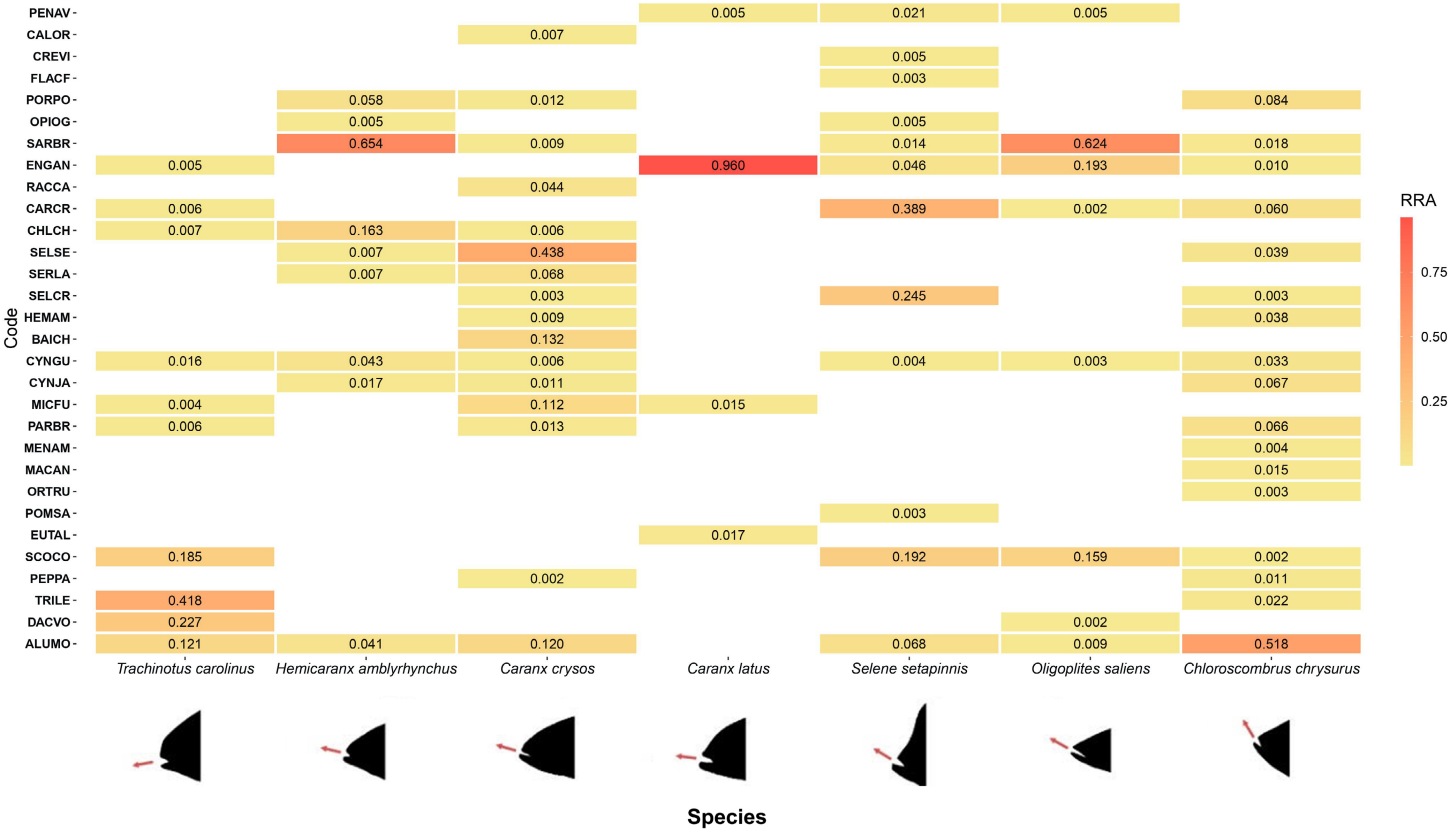
Relative read abundance heatmap for the principal dietary items in seven carangid fishes caught as bycatch in the Southern Brazilian Sardine fishery. Dietary items are phylogenetically ordered and coded as in Ready et al. (2023). Carangids are ordered based on the relative position of the mouth from left (subterminal/terminal) to right (superior) as indicated by red arrows. Prey species: PENAV, *Penilia avirostris*; CALOR, *Callinectes ornatus*; CREVI, *Creseis virgula*; FLACF, *Flaccisagitta aff. enflata*; PORPO, *Porichthys porosissimus*; OPIOG, *Opisthonema oglinum*; SARBR, *Sardinella brasiliensis*; ENGAN, *Engraulis anchoita*; RACCA, *Rachycentron canadum*; CARCR, *Caranx crysos*; CHLCH, *Chloroscombrus chrysurus*; SELSE, *Selene setapinnis*; SERLA, *Seriola lalandidorsalis*; SELCR, *Selar crumenophthalmus*; HEMAM, *Hemicaranx amblyrhynchus*; BAICH, *Bairdiella chrysoura*; CYNGU, *Cynoscion guatucupa*; CYNJA, *Cynoscion jamaicensis*; MICFU, *Micropogonias furnieri*; PARBR, *Paralonchurus brasiliensis*; MENAM, *Menticirrhus americanus*; MACAN, *Macrodon ancylodon*; ORTRU, *Orthopristis ruber*; POMSA, *Pomatomus saltatrix*; EUTAL, *Euthynnus alletteratus*; SCOCO, *Scomber colias*; PEPPA, *Peprilus paru*; TRILE, *Trichiurus lepturus*; DACVO, *Dactylopterus volitans*; ALUMO, *Aluterus monoceros*.

The overall dataset for the analysis shows saturation in the number of MOTUs recovered per sample with increasing sequencing coverage (Ready et al., 2023) and recovery of all the same prey taxa using clustering at the lower level of 97%. Negative controls presented very low read counts and very few reads of common contaminant taxa. Less than 10 reads were found in any given sample of human DNA and other species being worked on elsewhere by the research groups involved, indicating that our decontamination procedures were successful. High read counts for the MOTU assigned to each carangid fish confirmed the taxonomic identities originally assigned to the individuals from which the stomach contents originated. After the rule‐based automated data cleaning and manual curation, a few remaining MOTUs were considered to represent likely secondary consumption within each species and removed from further analysis. Only a few dietary items were identified that were also caught in the same net as the fish whose stomach contents were analyzed. These were only maintained as probable dietary items when the RRA of the item was greater than that of other items which were not caught in the same net as the same sample. Almost all these items were also identified in the diet of individual samples that were not collected from the same net. Additionally, although *Sardinella brasiliensis* (Steindachner, 1879) which is the target of the fishery was found in most sampling events, it was maintained in analyses as it is a common dietary item identified in previous studies of these species. A final table of accepted prey items was produced that included taxa belonging to four phyla, 12 orders, 17 families, 29 genera, and 30 species (Ready et al., 2023) and the composition of the diet of each of the seven species visualized in a heatmap (Figure 1).

**FIGURE 2:**
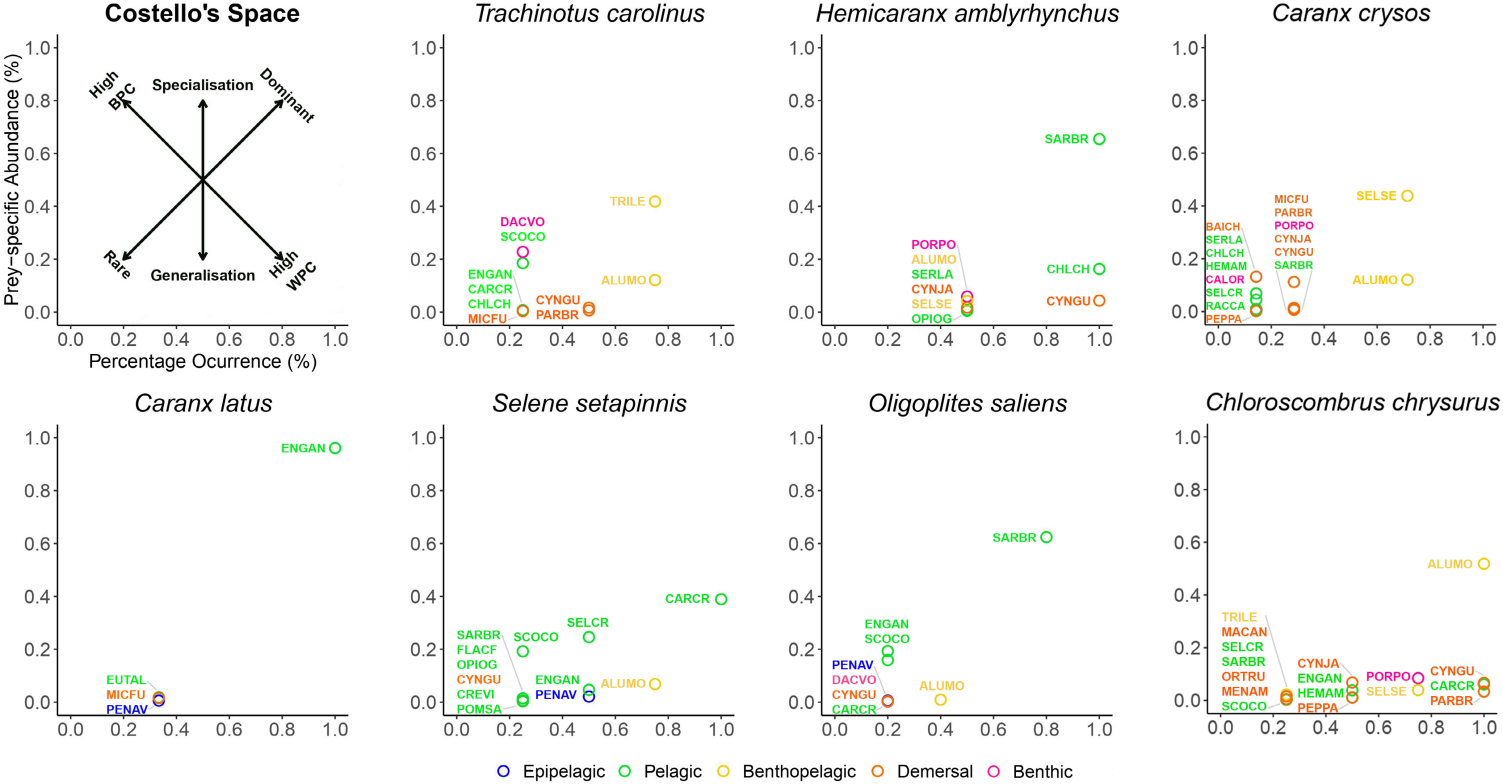
Costello graphs showing the role of different prey items in the diet of seven species of Carangid fishes caught as bycatch from the Southern Brazilian Sardine fishery, highlighting the change in dominance of prey items from different layers in the water column. Prey species: PENAV, *Penilia avirostris*; CALOR, *Callinectes ornatus*; CREVI, *Creseis virgula*; FLACF, *Flaccisagitta aff. enflata*; PORPO, *Porichthys porosissimus*; OPIOG, *Opisthonema oglinum*; SARBR, *Sardinella brasiliensis*; ENGAN, *Engraulis anchoita*; RACCA, *Rachycentron canadum*; CARCR, *Caranx crysos*; CHLCH, *Chloroscombrus chrysurus*; SELSE, *Selene setapinnis*; SERLA, *Seriola lalandidorsalis*; SELCR, *Selar crumenophthalmus*; HEMAM, *Hemicaranx amblyrhynchus*; BAICH, *Bairdiella chrysoura*; CYNGU, *Cynoscion guatucupa*; CYNJA, *Cynoscion jamaicensis*; MICFU, *Micropogonias furnieri*; PARBR, *Paralonchurus brasiliensis*; MENAM, *Menticirrhus americanus*; MACAN, *Macrodon ancylodon*; ORTRU, *Orthopristis ruber*; POMSA, *Pomatomus saltatrix*; EUTAL, *Euthynnus alletteratus*; SCOCO, *Scomber colias*; PEPPA, *Peprilus paru*; TRILE, *Trichiurus lepturus*; DACVO, *Dactylopterus volitans*; ALUMO, *Aluterus monoceros*.

Considering this dataset for principal prey items, teleost fishes were the most common prey with 99.73% of reads, while invertebrates represented the remaining 0.27% of reads of accepted prey items. This difference may reflect true consumption patterns but also likely reflects amplification bias of chordates due to the primers used to amplify the COI fragment as well as a more complete reference database for chordates. Between four (*C. latus*) and 17 (*C. chrysurus*) dietary items were identified, with the total number of prey items generally correlated with the number of individuals sampled. This suggests that increased sample numbers will be important to describe the full variation in the diet when using metabarcoding approaches. The large number of fish species consumed and the reciprocal consumption between carangids (either as dominant items, as shown by high RRA, in the reciprocal consumption between *C. crysos* and *S. setapinnis* or as less important items in the reciprocal consumption between *C. crysos* and *C. chrysurus*) is interpreted as indicative of the importance of early life stages (eggs, larvae or small juveniles) as prey in the diet of many of these species (Figures 1 and 2, Ready et al., 2023).

Invertebrate prey items were only found in the diet of some carangids and always at low relative abundance. Pelagic invertebrates (a branchiopod crustacean—*Penilia avirostris*, a pteropod mollusk—*Creseis virgula* and a chaetognath—*Flaccisagitta aff. enflata*) were found in the diet of *S. setapinnis*, with *P*. avirostris also found in the diet of *C. latus* and *O. saliens*. The only benthic invertebrate identified was the swimming crab *Callinectes ornatus*, found in the diet of *C. crysos*. Adult *C. ornatus* are relatively large (Mantelatto & Fransozo, 1997) in comparison to the individual of *Caranx crysos* found to consume it. This, combined with the low relative abundance, suggests that this crab is a low volume item in the diet, probably as a small individual such as a larval stage. The failure to identify more benthic‐limited and non‐fish prey items in the diet may reflect true consumption preferences for fish as prey, or amplification bias, or a lack of identification of invertebrate taxa because of limited reference sequences for the region's fauna. *S. setapinnis* has recently been described to be piscivorous but with zooplankton complementing the diet while *C. chrysurus* were found to consume copepods and larval crustaceans including luciferids (Olher & Gasalla, 2023). The luciferid shrimp *Belzebub faxoni* was identified in the raw sequence data, but with very low read counts (maximum of 31 reads in a single sample, CLA5, that contained >30,000 reads of potential prey items) and therefore filtered out as a probable contaminant. Considering that PCR is known to be difficult for crustacean taxa (Machida et al., 2004), amplification bias may strongly affect the successful identification of crustacean taxa as significant components in dietary studies. Dietary diversity does not obviously correlate with variability in size of individuals sampled. Despite greater variation in individual sizes in the species *O. saliens* and *T. carolinus* they show either high or low diversity in their diets, respectively, while species with minimal size variation such as *C. crysos* and *S. setapinnis* show moderate to high diversity in their diets (Figure 1, Ready et al., 2023).

**FIGURE 3:**
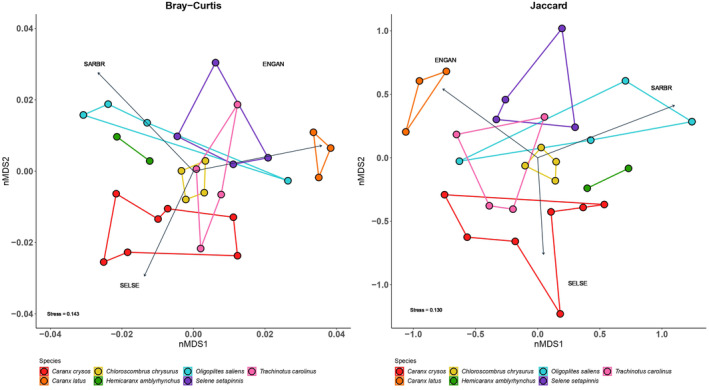
Non‐metric multidimensional scaling (nMDS) showing the variation in the diet of seven species of Carangid fishes caught as bycatch from the Southern Brazilian Sardine fishery. The similar (mirrored) pattern between analyses based on read abundance (Bray–Curtis) and presence (Jaccard) supports consistency of the data for dietary interpretation. The main prey items as determined by the *envfit* routine (*p* < .05) are shown as arrows indicating the contribution of those items to placement in relation to the axes. Main contributing prey items: ENGAN, *Engraulis anchoita*; SARBR, *Sardinella brasiliensis*; SELSE, *Selene setapinnis*.

Although there is generally considerable overlap in the diet of many of the sampled taxa, Costello plots (Figure 2) and nMDS ordination analyses (Figure 3) indicate that, even with limited sampling numbers, a trend can be seen where foraging behavior is inferred to be associated with distinct use of habitats and the water column by these carangid species. The orientation of the mouth is generally associated with different and specific foraging patterns or environmental exploration (Abdussamad et al., 2013). The susceptibility of different prey species to superior, lateral, or inferior predatory attacks may reduce the trophic niche overlap between these species despite sharing opportunistic generalist feeding patterns (Selleslagh & Amara, 2015). In Figures 2 and 3, two of the three species that show superior opening mouths (*S. setapinnis* and *O. saliens*) fed mainly on fish with similar morphological patterns and found in pelagic environments. Specifically, they fed on other carangids and clupeiformes as found in some previous trophic analyses of these species (Carvalho & Soares, 1997; Sazima, 1998). Consumption of other dietary items from the pelagic layer has also been observed in other studies on the use of habitat by *S. setapinnis* (Cervigón et al., 1992; Olher & Gasalla, 2023) and *O. saliens* (Cervigón, 1993; Pichler et al., 2017). In contrast, the third species with a superior opening mouth, *C. chrysurus*, shows a more generalist diet (including predominance of the generally benthic associated taxa *Aluterus monoceros* and *Porichthys porosissimus*) as also observed in other studies (Cervigón, 1966; Cunha et al., 2000; Menezes & Figueiredo, 1980). Although *C. chrysurus* has a superior opening mouth, it swims at almost all depths and has been cited as a demersal fishing resource, depending on the region in which it is found (Cunha et al., 2000).

*C. crysos*, *C. latus*, and *H. amblyrhynchus* all have approximately terminal opening mouths (Abdussamad et al., 2013). Prey items identified for *C. crysos* are consistent with existing knowledge about the species' diet that describes it as an opportunistic predator consuming mainly pelagic organisms, but with benthic prey representing part of its diet (Sley et al., 2009). *C. latus* is also believed to explore the pelagic region (Claro, 1994; Peebles, 2004; Pichler et al., 2017; Smith‐Vaniz, 1986), and the consumption of the pelagic prey *E. anchoita* confirms previous knowledge (Velasco‐Reyes et al., 2022). Despite only processing two samples of *H. amblyrhynchus*, prey items were identified that included both pelagic and benthic/demersal species. This suggests that the pelagic diet cited in the literature (Cervigón, 1993) does not describe the full dietary range of this species. Even considering the role of pelagic life stages of benthic/demersal species, the presence of *Porichthys porosissimus* (Cuvier, 1829) in the diet (and also not caught in the same net) suggests that feeding does take place low in the water column, as species of *Porichthys* are nest guarders with larvae that tend to stay near the substrate at least during the day only swimming up a little to feed on zooplankton during the night (Arora, 1948).

The subterminal mouth of *T. carolinus* is consistent with the predominant consumption of prey from the benthopelagic to demersal strata (particularly *Trichiurus lepturus*, *Aluterus monoceros*, and *Dactylopterus volitans*—Figure 2), corroborating the results found in traditional studies of the diet of this species (Bellinger & Avault Jr, 1971; Palmeira & Monteiro‐Neto, 2010; Zahorcsak et al., 2000). In addition, *T. carolinus* has a well‐developed pharyngeal plate which is well‐suited for crushing prey items in the environment exploited by this species (Bellinger & Avault Jr, 1971), suggesting that unidentified invertebrate prey (either resulting from amplification bias or a lack of reference sequences) may also contribute to the diet.

The results obtained here support the partial trophic niche segregation of carangid fishes caught in the region. It is inferred that this results from a combination of differential use of the water column and feeding behavior associated with mouth morphology. However, partial overlap in diet may also be explained by the importance of maintaining a diverse diet to maximize energy density levels through continual consumption. The importance of a diversity of smaller prey items, especially early life stages of other fishes (as interpreted from the inter‐predation between *C. crysos* and *S. setapinnis* and other taxa) as well as invertebrates, are important areas for further investigation. Many of the prey items identified here using the metabarcoding approach will often digest quickly and are unlikely to be easily identified using traditional morphological methods, but using multiple markers (Berry et al., 2015) and data merging techniques (Burian et al., 2022) are important factors that can help further resolve diet in metabarcoding studies.

## 4 CONCLUSION

Metabarcoding has proven to be effective in refining the taxonomic resolution and recovering species level identities of a larger number of taxa in the diet of carangid fishes from the south‐west Atlantic. This has considerably improved knowledge on the trophic role of these fishes in comparison to previously published data. It has allowed identification of inter‐predation, a tendency toward partial trophic niche segregation associated with predatory behavior, morphology and use of the water column, and the probable importance of early life stages of other fishes as food sources. These findings raise significant questions about how dietary interactions are modeled in these ecosystems and will require consideration for future ecosystem‐based management of fisheries. Broader application of metabarcoding in the study of fish dietary analysis will require improved reference datasets, use of markers that reliably amplify invertebrate taxa, increased sampling numbers and seasonal sampling to better describe the relative contribution of diverse food sources but will provide insights that were unattainable using previous methods. These novel insights will help to provide complementary data for evidence‐based management.

## AUTHOR CONTRIBUTIONS

**Fabricio dos Anjos Santa Rosa:** Formal analysis (equal); investigation (equal); methodology (equal); visualization (lead); writing – original draft (equal); writing – review and editing (equal). **Maria A. Gasalla:** Conceptualization (equal); funding acquisition (equal); project administration (equal); resources (equal); supervision (equal); writing – review and editing (equal). **Anna Karolina Oliveira de Queiroz:** Data curation (equal); investigation (equal); methodology (equal); writing – original draft (equal); writing – review and editing (equal). **Talita Fernanda Augusto Ribas:** Data curation (equal); methodology (equal); writing – review and editing (supporting). **Quentin Mauvisseau:** Data curation (supporting); methodology (equal); resources (equal); writing – original draft (supporting); writing – review and editing (equal). **Hugo J. de Boer:** Funding acquisition (equal); resources (equal); supervision (equal); writing – review and editing (equal). **Birgitte Lisbeth Graae Thorbek:** Data curation (equal); investigation (equal); writing – review and editing (supporting). **Renato Renison Moreira Oliveira:** Data curation (equal); formal analysis (equal); resources (supporting); software (equal); writing – review and editing (supporting). **Marcele Laux:** Data curation (equal); formal analysis (equal); resources (supporting); software (equal); writing – review and editing (supporting). **Felippe A. Postuma:** Data curation (equal); investigation (equal); resources (supporting); writing – review and editing (supporting). **Jonathan Stuart Ready:** Conceptualization (equal); data curation (equal); funding acquisition (equal); methodology (equal); project administration (equal); resources (equal); supervision (equal); writing – original draft (equal); writing – review and editing (equal).

## CONFLICT OF INTEREST STATEMENT

The authors declare to have no conflicts of interest.

## Data Availability

DNA sequences: GenBank Study accession ID SRP269542 (with sample codes as indicated in Table 1). Sampling locations and metadata and all R scripts associated to this manuscript are as indicated in the (Ready et al., 2023) dataset Dryad DOI: https://doi.org/10.5061/dryad.cnp5hqc7w.
